# Correlations between biological markers of the perirenal adipose tissue and clinical features of patients with localized kidney cancer

**DOI:** 10.3389/fmed.2025.1676630

**Published:** 2025-11-03

**Authors:** Leonardo Rafael Romeo, Matías Nuñez, Matías Ferrando, Constanza Matilde López-Fontana, Rubén Walter Carón, Flavia Alejandra Bruna, Virginia Pistone-Creydt

**Affiliations:** ^1^Instituto de Medicina y Biología Experimental de Cuyo (IMBECU), Centro Científico y Tecnológico Mendoza, Consejo Nacional de Investigaciones Científicas y Técnicas (CONICET), Universidad Nacional de Cuyo, Mendoza, Argentina; ^2^Urocuyo, Centro de Urología y Transplante, Mendoza, Argentina; ^3^Instituto de Investigaciones en Biodiversidad y Medioambiente (INIBIOMA), Consejo Nacional de Investigaciones Científicas y Técnicas (CONICET), San Carlos de Bariloche, Argentina; ^4^Centro de Investigaciones Odontológicas (CIO), Facultad de Odontología, Universidad Nacional de Cuyo, Mendoza, Argentina; ^5^Departamento de Fisiología, Facultad de Ciencias Médicas, Universidad Nacional de Cuyo, Mendoza, Argentina

**Keywords:** human adipose tissue, renal cancer, epithelial-stromal interactions, machinelearning, leptin, adiponectin

## Abstract

Among the different types of cells that surround renal epithelial cells, human renal adipose tissue (hAT) is one of the most abundant. We have previously characterized the expression of different proteins in hAT (adiponectin, adiponectin receptor 1, leptin, leptin receptor, perilipin 1, and metalloprotease (1). In this study, we evaluated if the differential proteins expression as a whole was sufficient to separate healthy patients from patients with kidney cancer, using unsupervised machine learning algorithms; and the correlation between adiponectin and leptin expression with clinical characteristics of kidney cancer patients. Considering the six biological variables evaluated in the different hAT fragments, we were able to separate healthy from kidney tumor patients by unsupervised machine learning algorithms projection. In addition, a decrease in adiponectin expression was found in patients with a more undifferentiated tumor as well as in patients with a history of smoking. Also, there was a positive correlation between leptin, tumor size and difficulty in tumor dissection. The parameters that increase the difficulty in dissection are male sex, smoking history, tumor size and the fat striation degree in imaging studies. Moreover, PAT (perirenal adipose tissue)-related adipokine signatures reflect systemic metabolic dysfunction, including features of metabolic syndrome, offering additional value for anticipating surgical complexity and refining prognostic stratification. This project represents a new way of looking at kidney cancer, by correlating clinical features with specific biomarkers, we may be able to identify patterns that might predict how the disease will develop. This could lead to more accurate prognoses and more effective treatments.

## Introduction

Renal cell carcinoma (RCC) is among the top 10 most frequently diagnosed cancers globally. More than 300,000 patients are diagnosed with kidney cell cancer every year, and approximately 140,000 die from this disease (1). The RCC is characterized by its high metastatic potential, with around 30% of patients presenting with metastases at the time of diagnosis (2). The predominant RCC subtype is clear cell renal cell carcinoma (ccRCC), representing 80%−90% of all RCC cases. Despite recent improvements in survival rates for ccRCC patients, the global incidence of this cancer has been rising steadily at a rate of 2-3% annually. This trend may be linked to increasing rates of obesity and hypertension worldwide—key factors contributing to RCC development (3). Currently, surgical removal of the tumor remains the standard treatment for localized RCC. On the other hand, patients with metastatic or inoperable RCC typically receive systemic therapies involving targeted drugs and/or immune checkpoint inhibitors (4, 5). In addition to the epigenetic and genetic changes that occur in epithelial cells during the tumorigenic process, in recent years it has been shown that tumor progression also depends on the bidirectional dialogue between tumor epithelial cells and surrounding stromal cells. The kidney is surrounded principally by adipose tissue (AT). The AT is a bioactive endocrine organ (6, 7) that not only secretes soluble factors but also contributes significantly to the composition of the extracellular matrix (ECM). In obesity and adipose tissue expansion, hypoxia and inflammation stimulate adipocytes and stromal cells to secrete ECM components such as collagens (particularly types I, III, and VI), fibronectin, and matrix metalloproteinases (8, 9). This ECM deposition, often excessive and disorganized, contributes to adipose tissue fibrosis, mechanical stiffness, and impaired metabolic and vascular function—features increasingly recognized in visceral fat depots including PAT (10). Visceral adipose tissue (VAT), a hallmark of metabolic syndrome (MetS), plays a central role in systemic inflammation and tumor-promoting metabolic dysregulation. Among visceral fat depots, PAT is uniquely positioned to exert direct paracrine effects on renal tumors due to its anatomical proximity, while also influencing surgical outcomes through local inflammation and fibrosis (11). This dual role—metabolic and mechanical—makes PAT a particularly relevant target in the study and management of RCC. Dysfunctional behavior of the AT, often observed in obesity, has been widely appreciated as one of the main underlying causes of cancer (12). Factors secreted by peritumoral AT, ECM components, and direct cell-cell contact can influence the phenotypic behavior of malignant cells (13). For example, the increase in the local production of adipocytokines and the thickness of the fat surrounding the organ could be decisive for the progression and aggressiveness of the tumor (14–16). Invasion of the surrounding AT by tumor cells is a common pathological finding and a factor related to a worse prognosis. In fact, there is evidence showing that AT is essential in determining whether cancer cells progress toward metastasis, probably due to alterations in their metabolic activity and in the secretion of adipocytokines (17, 18). Our research group recently demonstrated that proteins secreted by adipose tissue interact with tumor cells via a paracrine mechanism. Furthermore, we have shown that the proximity of adipose tissue to cancer cells, alters the structure, function and protein expression profile of the peritumoral adipose tissue. Specifically, peritumoral adipose tissue (hRAT) expresses a higher amount of leptin and its receptor ObR, and a lesser amount of adiponectin, AdipoR1 (adiponectin receptor 1), the metalloprotease ADAMTS1 and perilipin 1 compared with normal perirenal adipose tissue (hRAN) (19–23).

Perirenal adipose tissue (PAT) is a type of visceral fat situated around the kidneys and adrenal glands, occupying the perirenal space between the renal capsule and Gerota's fascia (24). Structurally, PAT comprises a blend of white and brown fat cells, most of which remain inactive (25). It has been shown that adipose tissue produces and releases various adipokines and inflammatory molecules, such as adiponectin, leptin, visfatin, resistin, TNFα, IL-6, and IL-1β. These factors significantly influence numerous cellular mechanisms through autocrine, paracrine, and endocrine pathways (26). Research has repeatedly demonstrated a link between PAT thickness and the progression of kidney disorders. Obesity, for instance, is often associated with proteinuria, and individuals with a BMI (Body Mass Index) exceeding 25 are three times more likely to develop chronic kidney disease (CKD). Leptin is thought to promote tumor growth, while adiponectin may have tumor-suppressing properties (27, 28). Additionally, leptin has been shown to support angiogenesis, whereas adiponectin can trigger apoptosis in tumor cells (29–32).

Over the last few years, numerous researchers have expressed a significant interest in the clinical study of perirenal fat. Specially, from a surgical point of view, there are patients with renal cancer who present greater adherence of the perirenal fat that makes the surgical dissection of the tumor difficult (33, 34). This can be predicted with imaging studies such as computed tomography (CT) or magnetic resonance imaging (MRI). In fact, a scale was proposed to classify the stranding affectation of the perirenal tissue based on image, that usually correlates to the difficulty of the surgical dissection (35). Other factors such as gender, smoking, body mass index and high blood pressure can also have an effect on the adhesion of fat (34). The clinical impact of PAT hypertrophy is mediated through a cascade of mechanical and metabolic dysfunctions. As PAT expands within the confined space of the renal fascia, extrinsic compression of its intrinsic microvasculature occurs. This impairs local blood flow, leading to regional hypoxia and ischemia within the adipose depot. Hypoxia is a well-established driver of adipose tissue dysfunction, primarily via stabilization of hypoxia-inducible factor-1 alpha (HIF-1α), which activates a transcriptional program that promotes macrophage infiltration, inflammation, and fibrosis in white adipose tissue (8, 36). In this hypoxic microenvironment, adipocytes shift toward a pro-inflammatory phenotype, secreting elevated levels of cytokines such as TNF-α, IL-6, and MCP-1, while anti-inflammatory adipokines like adiponectin are suppressed (22). This chronic low-grade inflammation facilitates the recruitment of fibroblasts and the deposition of extracellular matrix components, such as collagen and fibronectin, contributing to tissue fibrosis (37). In perirenal fat, this fibrotic remodeling obliterates the natural avascular plane between the renal capsule and Gerota's fascia, making surgical dissection substantially more difficult. Clinically, this phenomenon is recognized as adherent perinephric fat (APF), which has been associated with prolonged operative time, increased intraoperative bleeding, and higher technical complexity during nephron-sparing surgery (38). Histopathological studies have also demonstrated that APF is characterized by enlarged adipocytes and increased collagen content, confirming the fibrotic and inflammatory transformation of perirenal fat (39). Thus, radiologic findings such as perinephric stranding or hypertrophy are not merely incidental but are indicative of a pathophysiological process that bridges metabolic dysfunction, local tissue inflammation, and surgical morbidity. Clinical and epidemiological data of patients who undergo partial or total nephrectomies, and from which we obtain the adipose tissue fragments that surround the kidney tumor to advance our studies, are carefully recorded. In this work, (1) we evaluate the correlation between adiponectin and leptin expression with clinical and pathological characteristics of kidney cancer patients, and (2) we evaluated whether differential expression of hAT-secreted proteins was sufficient to separate healthy patients from patients with kidney cancer, using unsupervised machine learning algorithms. We hypothesize that the differential expression profile of human adipose tissue-secreted proteins is sufficient to distinguish cancer patients from healthy through unsupervised machine learning analysis, and that alterations in adipokine expression (specifically decreased adiponectin and increased leptin) are associated with adverse clinical and pathological features in kidney cancer patients.

## Method

### Reagents

Reagents were from Sigma Chemical Co (St. Louis, MO, USA), cell and tissue culture materials from Falcon Orange Scientific (Graignette Business Park, Belgium), and culture media from both tissue and cell lines and supplements were from Gibco BRL (Carlsbad, CA, USA).

### Sample collection and handling

Explants of human perirenal adipose tissue were taken as follows: (1) in the case of patients with renal tumor, a fragment of adipose tissue close to 1 cm from the tumor kidney was taken (hRAT; *n* = 32); (2) in the case of living kidney donors, the adipose tissue fragment was taken 1 cm away from the kidney, using the middle zone (middle pole) of geographical reference. The selected patients with kidney cancer had not received previous chemotherapy or radiotherapy treatment. Trying, in all cases, to take the sample 1 cm from the location of the tumor, getting as close as possible to the middle zone. In all cases, biopsies were taken distant from the adrenal gland (sources of norepinephrine).

The BMI of patients was: 29.16 kg/m^2^ for patients with renal tumor (hRAT), and 25.78 kg/m^2^ for living kidney donors (hRAN). BMI (kg/m^2^) was calculated as weight (kg) divided by height (m) squared.

hRAN y hRAT samples were transported in PBS and processed immediately. On average, 2 h elapsed from the acquisition of the surgical sample until it was processed under a sterile laminar flow hood. The project was approved by the Medical School's ethics committee (Universidad Nacional de Cuyo, Argentina). All patients gave their informed consent to undergo tissue harvesting for this research.

A fragment of each AT was used for qRT-PCR, Western blot and/or immunohistochemical studies, and the expression of adiponectin, AdipoR1, leptin, ObR, perilipin, and ADAMTS1 was measured (22).

### Kidney tumor patients

The following clinical characteristics of both, living kidney donors and patients with renal tumor were recorded: sex, age, BMI, and smoking history. Also, pathological and surgical characteristics of patients with ccRCC were taken: degree of tumor differentiation, size of the lesion (T), the density of this adipose tissue measured by degree of striation (standing of Mayo adhesive score) in computed tomography (CT) performed prior to surgery, and the difficulty in the surgical dissection of the tumor by separating it from adipose tissue (Supplementary Figure 1). This last information was evaluated by two surgeons and is classified as: 1- no adhesion; 2- moderate adhesion; 3- severe adhesion. Moreover, all surgeries were video recorded, and confirmation of this surgical scale was double checked.

### Unsupervised machine learning dimensionality reduction

To evaluate the relevance of proteins expressed by perirenal adipose tissue in defining a patient's health status, we sought to see how patients are grouped according to the status of the six proteins considered.

Thus, if each patient is represented by a vector in an six dimensional space where each coordinate represents the quantity of each the six proteins considered (adiponectin, AdipoR1, leptin, ObR, perilipin, and ADAMTS1) then the full set of patients is represented as that many points in the multidimensional space. Patients with a similar set of associated variables can be expected to be nearby points in this space. Hence, characterizing the full set of patients with the chosen proteins maps to the problem of identifying the neighborhoods of the set of vector points within the multidimensional space. For this purpose, the Uniform Manifold Approximation and Projection (UMAP) (40) dimension reduction algorithm is used as implemented in Tensorflow (41). Dimensionality reduction for visualization was performed with UMAP, using settings tailored for small samples. We set n_neighbors = 10 (=half the sample) to stabilize global structure, min_dist = 0.10 to avoid over-fragmentation while preserving cluster compactness, n_components = 2 with spectral initialization, and n_epochs = 5,000 to ensure convergence; the metric was euclidean. All remaining parameters were left at package defaults (e.g., learning_rate = 1.0, negative_sample_rate = 5, repulsion_strength = 1.0). This is a state-of-the-art unsupervised machine learning algorithm for dimension reduction based on manifold learning techniques and topological data analysis. It works by estimating the topology of high-dimensional data and uses this information to construct a low-dimensional representation that preserves the relationships present in the data. This technique is used to find clusters of patients that share similar health states.

### Statistical methods

The correlation between the expression of adiponectin and leptin with clinical characteristics of kidney cancer patients was evaluated using the SPSS Statistics program (SoftTonic, IBM). Results were considered significant at *p* < 0.05.

## Results

### Six proteins allow the identification of healthy patients from patients with renal tumors

**Figure 1:**
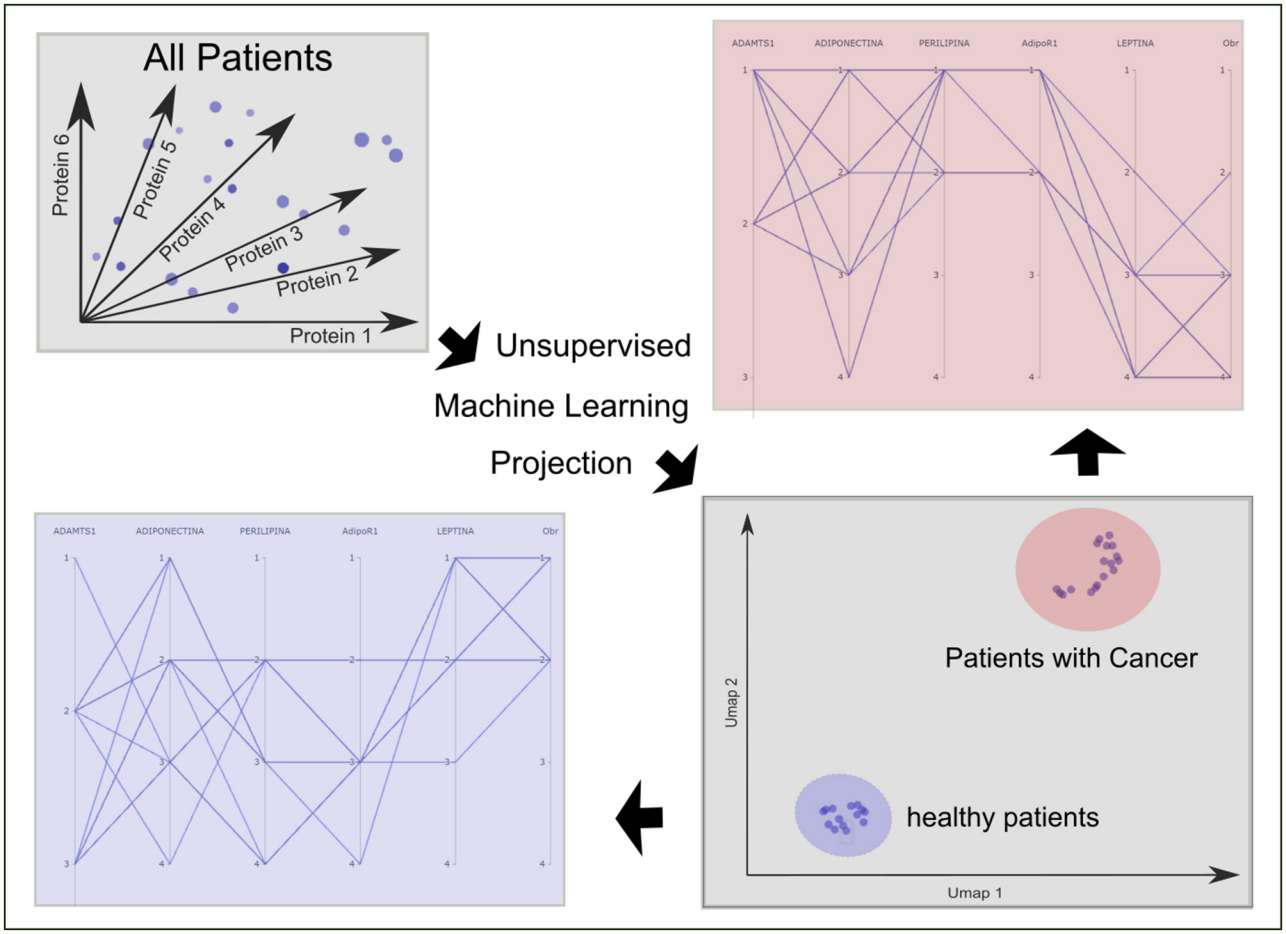
Relevance of proteins expressed by perirenal adipose tissue in defining the patient's health status. In order to evaluate how patients are grouped according to the level of expression of adiponectin, AdipoR1, leptin, ObR, perilipin, and ADAMTS1 we conducted an analysis of these six proteins by dimensional embedding from six dimensions to 2D two well-differentiated clusters of patients. Surprisingly, one of the clusters grouped healthy patients, while the other cluster grouped all patients with renal tumor. The result is robust as it was obtained both using UMAP and t-SNE.

The six proteins measured in AT fragments were adiponectin, AdipoR1, leptin, ObR, perilipin 1, and ADAMTS1 (22). In order to assess their relevance to identify healthy patients from patients with renal tumors, an unsupervised machine learning analysis was performed. The global analysis of the six proteins by dimensional embedding from six dimensions to 2D two well-differentiated clusters of patients. Surprisingly, one of the clusters grouped healthy patients, while the other cluster grouped all patients with renal tumor (Figure 1). The result is robust as it was obtained both using UMAP and t-SNE (https://github.com/lmcinnes/umap). The distribution of protein quantities in each set of patients can be seen in the parallel coordinates plots in Figure 1. The differences between the healthy patients and patients with a tumor is clear.

### Clinical, pathological, and surgical characteristics of the patients with biological molecules

Table 1 shows the clinical data and pathological characteristics of the tumors corresponding to the 30 patients with renal tumors who underwent partial or total nephrectomy. By studying the correlation between different variables, we found that the clinical parameters that predicted greater difficulty in tumor dissection were: (1) patients of male gender (*p* = 0.003), (2) smoking history (*p* = 0.048), (3) tumor size (*p* = 0.03), and (4) stranding on image (*p* = 0.001). No tumor invasion, toward the perirenal fat, was detected in any of the patients included in this study.

**Table 1:** Clinical data and pathological characteristics of the tumors corresponding to the patients with renal tumors.

**A**.	
**Clinical variables**	**%**
**Sex**	
Male	58.5
Female	41.5
**Age at diagnosis**	
< 50 years	18.5
50–59 years	22.2
60–69 years	22.2
>70 years	37.1
**Body mass index**	
< 18.5 kg/m^2^	0
18.5–24.9 kg/m^2^	18.5
25–29.9 kg/m^2^	37.1
≥30 kg/m^2^	44.4
**Smoking history**	
Yes	62.9
**B**.	
**Tumor variables**	***%***
**Degree of tumor differentiation**	
1	11.8
2	70.5
3	11.8
4	5.9
**Size of the tumor**	
1	70.4
2	18.5
3	11.1
**Difficulty in the surgical dissection**	
1	63.4
2	22.5
3	14.1
**Fat striation degree**	
1	59.2
2	27.3
3	13.5

Shows the values corresponding to 30 patients with kidney cancer of clinical characteristics (A) and pathological characteristics of the tumor (B).

Then, we studied possible correlations between the expression of adiponectin and leptin in the AT surrounding the renal tumor with the clinical and surgical characteristics evaluated in the patients:

### A decrease in adiponectin expression correlates with an increase in smoking and greater tumor undifferentiation

In order to evaluate if there are correlation among patients' clinical data of the patients and the biological variables assessed, we found that the decrease in the expression of adiponectin correlates with an increase in smoking habits (*p* = 0.046) and a greater tumor undifferentiation (*p* = 0.049; Table 2).

**Table 2:** Correlation between adiponectin and leptin with clinical and pathological characteristics of patients with kidney cancer.

**Correlated variables**	***r* value**	**Significance**	***N***
Adiponectin vs. tumor grade	−0.490	0.049	25
Adiponectin vs. smoking	−0.538	0.046	23
Leptin vs. size of the tumor	0.606	0.042	26
Leptin vs. difficulty in the surgical dissection	0.878	0.001	27
Difficulty in the surgical dissection vs. sex	0.512	0.003	27
Difficulty in the surgical dissection vs. size of the tumor	0.375	0.030	27
Difficulty in the surgical dissection vs. smoking	0.373	0.048	25
Difficulty in the surgical dissection vs. fat striation degree	0.470	0.007	27

A decrease in adiponectin expression was found in patients with a more undifferentiated tumor as well as in patients with a history of smoking. Also, there was a positive correlation between leptin, tumor size, and difficulty in tumor dissection. The parameters that increase the difficulty in dissection are male sex, smoking history, tumor size, and the fat striation degree in imaging studies.

### An increase in leptin expression correlates with a larger tumor size and a greater difficulty of surgical dissection

To determine whether there is a correlation between patients' socio-demographic data and the biological variables analyzed, we identified that an increase in leptin expression correlates with larger tumor size (*p* = 0.042) and greater difficulty of surgical dissection (*p* = 0.001; Table 2).

## Discussion

In tumor development and maintenance of a cancerous phenotype, bidirectional communication between epithelial cells and stromal environment is necessary. Among the different types of cells surrounding renal epithelial cells, renal adipose tissue is one of the most abundant. The adipose tissue is a bioactive endocrine organ that not only secretes soluble factors but also contributes significantly to the composition of the extracellular matrix (3). Cancer-associated adipocites (CAAs) significantly affect tumor growth, metastasis, and drug sensitivity via multiple mechanisms, including paracrine, juxtacrine and endocrine signaling, metabolites and metabolic reprogramming (42). We have previously shown that periprostatic adipose tissue from prostate cancer patients could influence tumor behavior even at early stages. Besides, we have worked with adipose tissue fragments from human breast tumors and normal mammary gland and demonstrated the importance of epithelial cell microenvironment, in particular of human adipose tissue, in the regulation of growth and metastatic capacity of human breast epithelial cells (19, 20). Recently, we demonstrated that renal peritumoral adipose tissue undergoes a process of adaptation to changes locally generated by the tumor (21, 22) and we show that this hRAT, already modified respect to hRAN, is capable of stimulating a protumorigenic behavior of renal epithelial cells. Moreover, we characterized the expression of different proteins in hRAT compared to hRAN.

In recent years, the use of machine learning has gained great importance as a way of visualizing high dimensional spaces and for establishing structures between data that allow predicting the behavior of some diseases (43). Thus, in this work we evaluate the relevance of six proteins (adiponectin, AdipoR1, leptin, ObR, perilipin, and ADAMTS1) expressed by perirenal adipose tissue in defining patient health. We found that by taking into consideration the concentration of these proteins, we were able to perfectly separate healthy patients from kidney cancer patients (Figure 1). This result not only confirms the differential behavior of normal perirenal adipose tissue (hRAN) compared to peritumoral adipose tissue (hRAT), but also the PAT could be used as a predictive organ/tool of the status of patients with kidney cancer. Specifically, we may incorporate the analysis of this protein profile of a fragment of perirenal AT patients in whom a surgery for renal cancer is going to be performed for a more accurate understanding of this disease. This is a promising new medical research area to investigate different types of diseases. High levels of inflammatory markers, biomarkers associated with tissue remodeling, might correlate with more invasive or fast-growing tumors, and could signal early metastatic potential (44). This could allow clinicians to stratify patients by risk, even before surgical intervention and even may enable closer monitoring and earlier intervention. Biomarkers could guide decisions regarding the need for adjuvant therapies, such as targeted therapies or immunotherapies. The different profiles obtained may help us to redefine patient conditions, as a prognostic tool, and perhaps decide which patients would benefit from adding adjuvant medical treatments after surgery.

Adipose tissue hypertrophy in visceral and peritumoral fat leads to local hypoxia, which triggers chronic low-grade inflammation and ECM remodeling, promoting epithelial-to-mesenchymal transition (EMT), tumor invasiveness, and the formation of pre-metastatic niches. Hypoxia-inducible factors (HIFs), upregulated under these conditions, facilitate metastasis through angiogenesis, ECM degradation, and immune cell recruitment (45). We recently demonstrated that peritumoral adipose tissue can induce EMT in human renal epithelial cells and stimulate angiogenesis (23, 46). The level of expression of adiponectin and leptin correlates with the difficulty of surgical dissection as well as the tumor differentiation.

While MRI remains the gold standard for detailed fat quantification—especially with techniques like proton density fat fraction (PDFF)—and ultrasound (US) offers accessible assessment of fat thickness and function, our study uses CT for specific clinical advantages. CT uniquely allows measurement of fat attenuation in Hounsfield units (HU) and detection of perirenal fat stranding, both central to our analysis. Fat attenuation reflects adipose tissue inflammation and remodeling, with lower HU indicating lipid-rich, less inflamed fat, and higher HU suggesting fibrosis or inflammation (47). CT is also the most reliable method for identifying perirenal fat stranding—seen as linear or reticular areas of increased attenuation—linked to inflammatory or edematous changes (48). Despite lacking functional data and involving radiation, CT's high spatial resolution, rapid imaging, and established role in oncologic evaluation make it ideal for assessing perirenal fat alterations in renal cell carcinoma (Supplementary Figure 1).

Our findings gain biological plausibility when viewed in the context of metabolic syndrome (MetS) and visceral adiposity. Perirenal adipose tissue (PAT), as a visceral fat depot, is now recognized as metabolically active, influencing systemic inflammation, insulin resistance, and renal dysfunction—even in individuals with normal BMI. This is important given evidence that normal BMI can mask high visceral fat and metabolic risk, underscoring the need for individualized assessment beyond BMI alone (49). PAT thickness has been independently linked to MetS markers, renal vascular resistance, and eGFR decline, regardless of body weight. Thus, changes in hRAT protein expression may reflect metabolic dysfunction tied to visceral fat activity, suggesting that tumor–adipose interactions are shaped by systemic metabolic status. PAT biomarkers may therefore serve not only as local indicators of tumor behavior but also as broader markers of metabolic risk in renal cancer.

PAT hypertrophy initiates a cascade of pathological changes beginning with local hypoxia caused by vascular compression within the confined perirenal space. This hypoxic environment triggers inflammation, promoting extracellular matrix ECM remodeling and fibrosis that result in increased surgical adhesions and operative complexity. Concurrently, altered adipokine signaling—characterized by elevated leptin and reduced adiponectin levels—exacerbates this process by enhancing pro-inflammatory and pro-fibrotic pathways, which may also contribute to a more aggressive tumor microenvironment and poorer oncologic outcomes. Among the limitations of our study, in addition to those already mentioned regarding BMI, are the sample size (healthy donors vs. patients), single-center nature, lack of longitudinal outcome data, and lack of routine assessment of systemic MetS measures (HOMA-IR, triglycerides, waist circumference).

This project represents a new way of looking at kidney cancer, by correlating clinical features with specific biomarkers, we may be able to identify patterns that might predict how the disease will develop. The characterization of Perinephric Adipose Tissue (PAT) pathology via non-invasive imaging markers, such as stranding and attenuation, provides immediate translational utility (50). Specifically, PAT biomarker profiling offers a simple, yet robust, tool to predict surgical difficulty by quantifying local inflammation and fibrosis preoperatively, thereby improving patient counseling and operative planning (50). Furthermore, by linking PAT characteristics to the aggressive tumor microenvironment, this approach refines existing prognostic stratification beyond traditional tumor staging (51). While large-scale prospective in a longitudinal cohort validation is still required to confirm these relationships, our findings strongly suggest that patients presenting with significant variations in these PAT biomarkers—even those with localized disease—may represent a distinct, high-risk subset. We hypothesize that integrating PAT profiles into risk assessment will be essential for identifying patients who could ultimately benefit from tailored adjuvant therapies, paving the way for a more comprehensive and personalized approach in renal cell carcinoma management. PAT biomarker profiling could be integrated into preoperative risk assessment and guide decisions on more careful dissection planning and adjuvant therapy selection, but prospective validation is required.

## Data Availability

The datasets presented in this study can be found in online repositories. The names of the repository/repositories and accession number(s) can be found below: The datasets generated during the current study are available in the CONICET repository, https://si.conicet.gov.ar/eva/bcoDatosInvestigacionLista.do?idModulo=9&idGrupo=9&idMenu=2535.
