# Supplementary figures and images for: Correlations between biological markers of the perirenal adipose tissue and clinical features of patients with localized kidney cancer
Source: Front Med (Lausanne). 2025 Nov 3;12:1676630. doi: 10.3389/fmed.2025.1676630 (PMC12620259; doi:10.3389/fmed.2025.1676630)

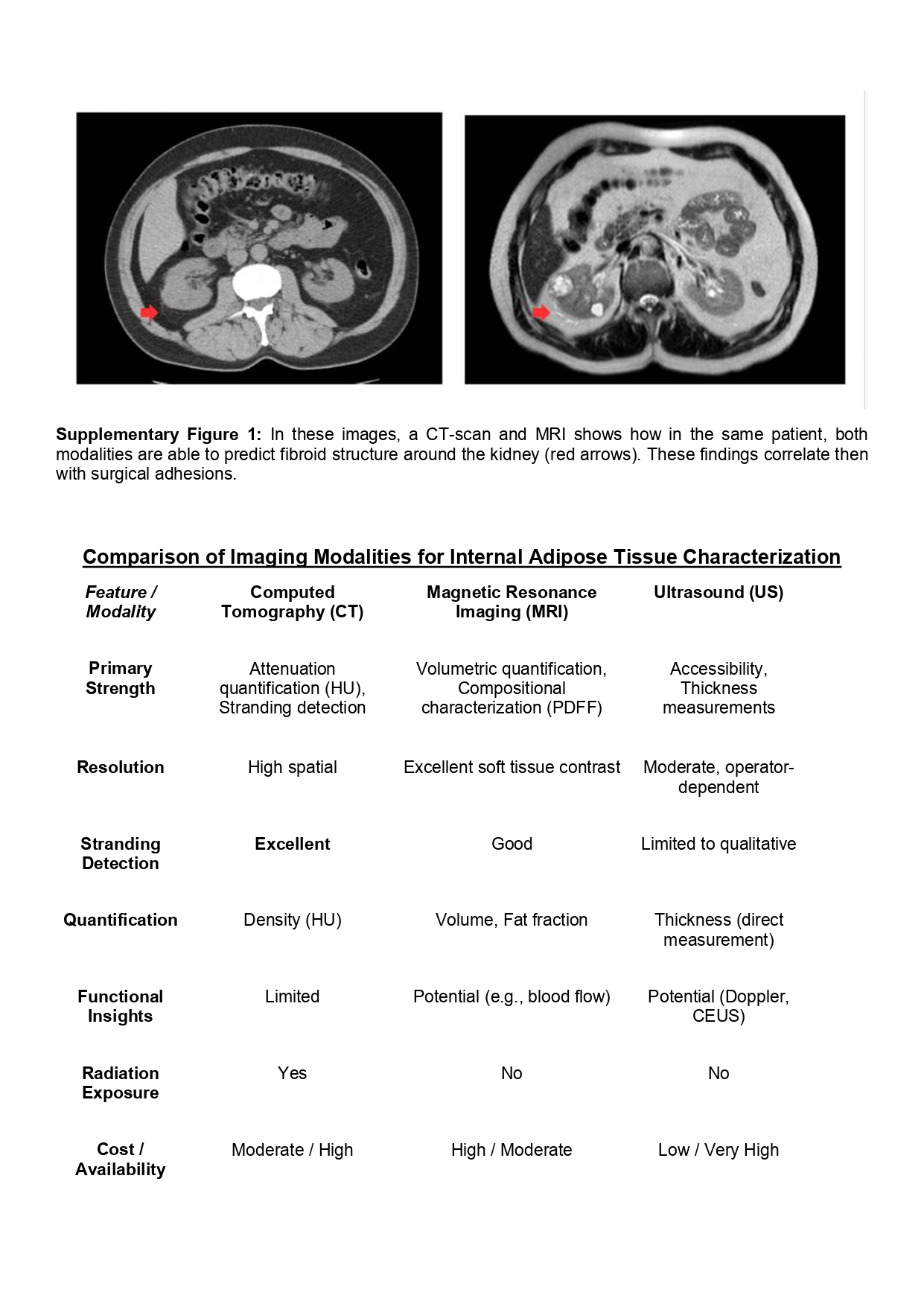

Supplement: Supplementary file 1 [file Image_1.jpeg]
